# A Dual-Stream Transformer with Self-Supervised Contrastive Training for fMRI-Based Autism Spectrum Disorder Classification

**DOI:** 10.3390/brainsci16030277

**Published:** 2026-02-28

**Authors:** Zirui Li, Lei Wang

**Affiliations:** Research Institute of Electronic Science and Technology, University of Electronic Science and Technology of China, Chengdu 611731, China; 202321230134@std.uestc.edu.cn

**Keywords:** Autism Spectrum Disorder, fMRI, time-series transformer, self-supervised learning, contrastive learning

## Abstract

**Background/Objectives**: Autism Spectrum Disorder (ASD) diagnosis is difficult due to heterogeneity. Current Time-series Transformer (TST) methods cannot capture both dynamic and global brain connectivity simultaneously, which limits ASD classification performance. **Methods**: We propose TwoTST, a dual-stream Transformer that combines raw Region of Interest(ROI) time series and Pearson correlation matrices(PCC).We pre-train the two TST branches via self-supervised learning by randomly masking ROIs and PCC, use contrastive learning and fine-tuning for feature alignment, evaluate five fusion strategies, and analyze relative parameter changes during fine-tuning. **Results**: Experiments were conducted on the ABIDE I dataset using the CC200 atlas. Contrastive learning, pre-training, and the dual-stream structure improve mean AUC by 3–6%, 3–7%, and 3–4% respectively. Attention Pooling is the optimal fusion strategy. Relative parameter changes are 0.32–0.44 for TST modules and 0.31–1.45 for contrastive projection heads. **Conclusions**: TwoTST effectively integrates dynamic and global connectivity for ASD identification. The proposed design outperforms single-stream models and provides a reliable approach for neuroimaging-based disorder classification.

## 1. Introduction

Autism Spectrum Disorder (ASD) is a neurodevelopmental disorder characterized by persistent deficits in social communication and interaction, along with restricted and repetitive patterns of behavior, interests, or activities [1]. ASD affects approximately 1 in 100 children globally [2], yet diagnosis remains challenging due to extensive phenotypic heterogeneity. Current diagnostic practices predominantly rely on behavioral assessments, which may be subjective and prone to observer bias [3], necessitating objective biomarkers for accurate and early diagnosis.

Functional magnetic resonance imaging (fMRI) has emerged as a powerful non-invasive tool for investigating brain function and connectivity patterns in ASD [4]. Resting-state fMRI (rs-fMRI) has shown promise for identifying neuroimaging biomarkers associated with ASD [5,6]. Previous studies have demonstrated the potential of using Pearson correlation coefficients computed from time series of regions of interest for classifying autism spectrum disorder via machine learning approaches [7,8,9]. However, these methods primarily focus on static functional connectivity patterns, treating correlation matrices as fixed representations of brain network topology, thereby neglecting the temporal dynamics inherent in fMRI data [10,11,12].

The advent of Transformer architectures has revolutionized time series analysis, thanks to their capacity to capture long-range temporal dependencies via self-attention mechanisms [13,14]. In neuroimaging, self-supervised pre-training has proven effective for Transformers to extract meaningful representations from fMRI data. Zhou et al. proposed masking entire ROIs in raw fMRI time series and reconstructing masked segments [15], while Mahler et al. developed pre-training by masking values in Pearson correlation coefficient matrices [16]. Both approaches demonstrated improved classification performance.

Despite these advances, existing Transformer-based fMRI analysis approaches face three key challenges: **Modality isolation:** most studies rely on a dichotomous approach, focusing either on temporal dynamics (raw ROI time series) or static functional connectivity (PCC matrices)—yet few have attempted to jointly model these two complementary modalities within a unified framework. **Feature misalignment:** though self-supervised pre-training has been explored to boost model generalization, few studies have designed pre-training objectives that explicitly align temporal and connectivity features in a shared representation space. Even when both modalities are integrated, their feature embeddings tend to remain isolated, leaving the inherent correlations between dynamic neural activity and stable functional connectivity unaccounted for.

To address these limitations, we propose TwoTST, a dual-stream Transformer framework that simultaneously captures dynamic temporal patterns and global functional connectivity from rs-fMRI data for ASD classification. **To address modality isolation**, we employ two specialized Transformer encoders: TST1 processes raw ROI time series for temporal dynamics, while TST2 processes PCC matrices for static connectivity. **To address feature misalignment**, we employ contrastive learning during the self-supervised pre-training stage to explicitly align the feature representations of the two Transformers, and evaluate five distinct fusion strategies in the fine-tuning phase to determine the optimal integration scheme. We further calculate the parameter change rate for both the dual-stream Transformer and the contrastive pre-trained model under the unfrozen fine-tuning setting, which validates the effectiveness of our pre-training strategy and the sensitivity of the proposed architecture. A comprehensive series of ablation experiments is conducted to verify the reliable classification performance of our framework. To assess the generalization capability of our model, we perform leave-one-site-out (LOSO) cross-validation across 19 sites on the dataset, demonstrate that our model possesses a certain degree of generalization ability across different fMRI acquisition sites. In addition, our model enables interpretability by identifying abnormal functional connections and atypical brain regions associated with ASD. Based on five independent runs with different random seeds, the optimal fusion strategy achieves a mean AUC of 0.74 ± 0.04. The code is available at https://github.com/Leezy-Ray/twoTST.

## 2. Related Work

### 2.1. fMRI-Based ASD Classification

Early approaches to ASD classification using fMRI data primarily relied on static functional connectivity measures, computing Pearson correlation coefficients between time series of different brain regions over entire scan durations [7,8]. Traditional machine learning classifiers, including support vector machines and random forests, have been applied to these static connectivity features with reasonable success [17,18]. However, these approaches fundamentally ignore the temporal dynamics inherent in fMRI data. The recognition that functional connectivity is inherently dynamic has led to the development of dynamic functional connectivity (dFC) methods [10,11,12], which employ sliding window techniques to compute connectivity matrices at different time points. While dFC methods have shown promise, they introduce challenges including the need for the careful selection of window parameters and increased dimensionality, which can exacerbate overfitting in small datasets [19].

Deep learning approaches have demonstrated potential for capturing complex patterns from high-dimensional brain data. Convolutional neural networks have been applied to functional connectivity matrices by treating them as images [20], while graph neural networks have been used to model the non-Euclidean structure of brain networks [21]. More recently, Transformer architectures have been adapted for neuroimaging applications, with hierarchical transformers proposed to learn inter-community relationships among brain regions [15]. However, most existing approaches focus on either temporal dynamics or static connectivity, rarely integrating both modalities effectively within a unified framework.

### 2.2. Self-Supervised Contrastive Learning and Multimodal Fusion

Self-supervised learning has emerged as a valuable paradigm for learning representations from limited labeled data in medical imaging [22]. Among self-supervised approaches, contrastive learning provides an effective way to align multimodal features by maximizing the similarity of consistent representations in a shared embedding space [23,24]. In fMRI analysis, masking-based pre-training strategies have demonstrated significant effectiveness. Zhou et al. proposed a framework that randomly masks entire ROIs in raw fMRI time series and reconstructs the masked segments [15], while Mahler et al. developed a multi-atlas enhanced Transformer framework that employs self-supervised pre-training by masking values in PCC matrices [16]. However, these approaches focus on single-modal pre-training, leaving the alignment between different modalities largely unexplored.

The integration of multiple data modalities has shown promise in improving classification performance in neuroimaging [25]. Different fusion strategies have been explored, including early fusion, late fusion, and intermediate fusion [26], with attention-based fusion mechanisms proving particularly effective [27]. However, most existing fusion approaches treat different modalities as relatively independent sources of information, without explicitly modeling the interactions and correlations between them. The development of fusion mechanisms that enable mutual interaction between different feature streams, particularly at the Transformer level, remains an underexplored area.

## 3. Materials and Methods

### 3.1. Framework Overview

**Figure 1:**
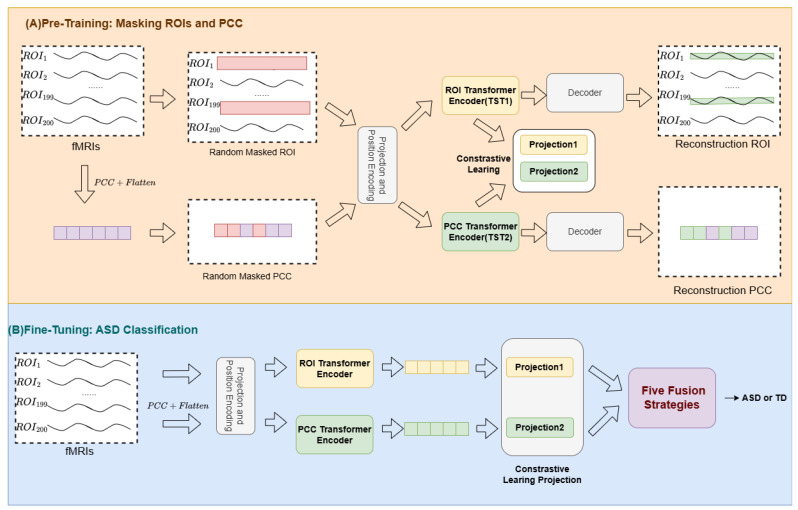
Overview of the TwoTST framework: (**A**) self-supervised contrastive pre-training with masked ROI and PCC reconstruction; (**B**) fine-tuning with five fusion strategies for ASD/TD classification.

We propose TwoTST, a dual-stream Transformer framework for ASD classification that integrates both dynamic temporal patterns and static functional connectivity from rs-fMRI data. As illustrated in Figure 1, our framework consists of two main phases: self-supervised pre-training and supervised fine-tuning.

In the pre-training phase (Figure 1A), both TST encoders are pre-trained independently using masked reconstruction. Given raw fMRI time series from R=200 ROIs, TST1 reconstructs masked ROI time series segments, while TST2 reconstructs masked PCC values computed from the time series. We then employ contrastive learning to train two linear projection heads. After pre-training, the TST encoders and the two linear projection layers are utilized for fine-tuning, during which we evaluate five fusion strategies and diverse parameter freezing settings for the pre-trained modules to identify the optimal configuration that yields the highest AUC.

In the fine-tuning phase (Figure 1B), the TST encoders extract features from ROI time series and PCC matrices, respectively. These extracted features go through the linear projection layers trained via contrastive learning and are then fused using five connection methods, namely Concat, Gated, Cross-Attention, Bilinear, and Attention Pooling (see Table 1).

### 3.2. Model Architecture

#### 3.2.1. ROI Transformer Encoder (TST1)

The ROI Transformer Encoder processes raw fMRI time series to capture temporal dynamics across brain regions. The input fMRI volumes are parcellated using the CC200 atlas into R=200 regions of interest (ROIs), resulting in a 2D matrix representation $X\in R^{T\times R}$, where *T* denotes the number of time points. Each time point is projected to a hidden dimension $d_{1}=512$ through a linear projection layer, and sinusoidal positional encodings are added to encode temporal order. A learnable [CLS] token is prepended to the sequence, which is then processed through $L_{1}$ Transformer encoder layers with multi-head self-attention, feed-forward networks, residual connections, and layer normalization. The final representation $h_{1}\in R^{d_{1}}$ is extracted from the [CLS] token output. During self-supervised pre-training, TST1 learns to reconstruct masked ROI time points. Specifically, we randomly mask $m_{1}\%$ of time points and train the encoder–decoder to reconstruct the masked values:

(1) $$L_{MSE}^{ROI}=\frac{1}{\left|M\right|}\sum_{(t,r)\in M}{\left(x_{r}^{t}-{\hat{x}}_{r}^{t}\right)}^{2}$$

where M denotes the set of masked time points, $x_{r}^{t}$ is the original ROI value, and ${\hat{x}}_{r}^{t}$ is the reconstructed value. This enables the encoder to capture dynamic temporal patterns from the raw time series.

#### 3.2.2. PCC Transformer Encoder (TST2)

The PCC Transformer Encoder processes functional connectivity matrices to capture global static patterns. Given the preprocessed fMRI time series $X\in R^{T\times R}$, we compute the Pearson correlation coefficient matrix $\mathrm{PCC}\in R^{R\times R}$:

(2) $$PCC_{ij}=\frac{\sum_{t=1}^{T}\left(x_{i}^{t}-{\bar{x}}_{i}\right)\left(x_{j}^{t}-{\bar{x}}_{j}\right)}{\sqrt{\sum_{t=1}^{T}{\left(x_{i}^{t}-{\bar{x}}_{i}\right)}^{2}}\cdot \sqrt{\sum_{t=1}^{T}{\left(x_{j}^{t}-{\bar{x}}_{j}\right)}^{2}}}$$

The upper triangular elements (excluding the diagonal) are extracted and flattened into a vector $p\in R^{D}$, where $D=\frac{R(R-1)}{2}=$ 19,900. The flattened vector is segmented into *N* patches, each embedded through a linear projection to dimension $d_{2}=256$. After adding positional encodings, the patches are processed through $L_{2}$ Transformer encoder layers with the same architecture as TST1. The final representation $h_{2}\in R^{d_{2}}$ is obtained via mean pooling and layer normalization across the patch dimension. During self-supervised pre-training, TST2 learns to reconstruct masked elements in the flattened PCC vector. Specifically, we randomly mask $m_{2}\%$ of elements in p and reconstruct them:

(3) $$L_{MSE}^{PCC}=\frac{1}{\left|M\right|}\sum_{d\in M}{\left(p_{d}-{\hat{p}}_{d}\right)}^{2}$$

where M denotes the set of masked positions, $p_{d}$ is the original PCC value, and ${\hat{p}}_{d}$ is the reconstructed value. This enables the encoder to capture global connectivity structures from the correlation matrix.

#### 3.2.3. Self-Supervised Pre-Training

Both encoders are pre-trained using a masked reconstruction objective. For TST1, we randomly mask $m_{1}\%$ of time points in the ROI sequences and train the encoder–decoder architecture to reconstruct the masked values. For TST2, we randomly mask $m_{2}\%$ of the PCC vector elements and reconstruct them. The pre-training loss is

(4) $$L_{pretrain}=L_{MSE}^{ROI}+\lambda L_{MSE}^{PCC}$$

where $L_{MSE}$ denotes mean squared error and λ is a balancing coefficient.

### 3.3. Contrastive Learning for Feature Alignment

To address the issue of feature misalignment between temporal and connectivity modalities, we introduce a contrastive learning strategy to align the feature representations from the two TST encoders in a shared embedding space. Let $h_{t}\in R^{d}$ denote the feature vector extracted by TST1 from ROI time series, and $h_{p}\in R^{d}$ denote the feature vector extracted by TST2 from the corresponding PCC matrix. These features are projected into a contrastive latent space via a shared linear projection head:

(5) $$z_{t}=\mathrm{proj}\left(h_{t}\right)=\mathrm{MLP}\left(h_{t}\right),\;z_{p}=\mathrm{proj}\left(h_{p}\right)=\mathrm{MLP}\left(h_{p}\right)$$

where proj(·) denotes the linear projection head (implemented as a two-layer MLP) and $z_{t},z_{p}\in R^{m}$ are the normalized projected features (${∥z∥}_{2}=1$). The InfoNCE contrastive loss is then minimized to pull positive pairs (features from the same subject) closer and push negative pairs (features from different subjects) apart:

(6) $$L_{\mathrm{CL}}=-\log\frac{\exp(\mathrm{sim}\left(z_{t},z_{p}\right)/\tau )}{\sum_{i=1}^{N}\exp\left(\mathrm{sim}\left(z_{t},z_{i}\right)/\tau \right)}$$

where $\mathrm{sim}\left(a,b\right)=a^{⊤}b$ is the cosine similarity, τ is the temperature parameter, and *N* is the number of negative samples in the batch.

### 3.4. Five Transformer-Level Fusion Strategies

To effectively integrate the complementary information from temporal dynamics (TST1) and functional connectivity (TST2), we evaluate five connect fusion methods. All methods first align features to a common dimension $d_{f}$ through linear projection:

(7) $${\tilde{h}}_{1}=W_{1}h_{1}+b_{1},\;{\tilde{h}}_{2}=W_{2}h_{2}+b_{2}$$

where $W_{1}\in R^{d_{f}\times d_{1}}$ and $W_{2}\in R^{d_{f}\times d_{2}}$.

#### 3.4.1. Concat

The simplest approach directly concatenates the aligned features:

(8) $$h_{fused}=\left[{\tilde{h}}_{1};{\tilde{h}}_{2}\right]\in R^{2d_{f}}$$

#### 3.4.2. Gated

This method employs learnable gating weights for adaptive fusion:

(9) $$g=\sigma \left(\mathrm{MLP}\left(\left[h_{1};h_{2}\right]\right)\right),\;h_{fused}=g⊙{\tilde{h}}_{1}+\left(1-g\right)⊙{\tilde{h}}_{2}$$

where σ is the sigmoid function and ⊙ denotes element-wise multiplication.

#### 3.4.3. Cross-Attention

Bidirectional Cross-Attention with residual connections enables mutual information exchange:

(10) $$c_{1}=\mathrm{CrossAttn}\left({\tilde{h}}_{1},{\tilde{h}}_{2}\right),\;c_{2}=\mathrm{CrossAttn}\left({\tilde{h}}_{2},{\tilde{h}}_{1}\right)$$

(11) $$h_{1}^{\prime }=\mathrm{LayerNorm}\left({\tilde{h}}_{1}+c_{1}\right),\;h_{2}^{\prime }=\mathrm{LayerNorm}\left({\tilde{h}}_{2}+c_{2}\right)$$

(12) $$h_{fused}=\mathrm{MLP}\left(\left[h_{1}^{\prime };h_{2}^{\prime }\right]\right)\in R^{d_{f}}$$

where $\mathrm{CrossAttn}\left(Q,K,V\right)=\mathrm{softmax}\left(QK^{⊤}/\sqrt{d_{f}}\right)V$.

#### 3.4.4. Bilinear

Bilinear transformation captures feature interactions with residual connection:

(13) $$h_{bilinear}=\mathrm{Bilinear}\left(h_{1},h_{2}\right),\;h_{residual}=W_{1}h_{1}+W_{2}h_{2}$$

(14) $$h_{fused}=\mathrm{LayerNorm}\left(h_{bilinear}+h_{residual}\right)$$

#### 3.4.5. Attention Pooling

Attention-weighted aggregation of the two features:   

(15) $$\alpha =\mathrm{softmax}\left(\mathrm{MLP}\left(\left[{\tilde{h}}_{1};{\tilde{h}}_{2}\right]\right)\right),\;h_{fused}=\alpha_{1}{\tilde{h}}_{1}+\alpha_{2}{\tilde{h}}_{2}$$

#### 3.4.6. Classification Head and Training Strategy

The fused features from all methods are passed through a three-layer MLP classifier with hidden dimensions [256, 64, 2] to produce the final classification output. During fine-tuning, both TST1 and TST2 encoders are frozen, and only the projection layers, fusion module, and classifier are trained. This strategy significantly reduces the number of trainable parameters, mitigating overfitting on the limited ASD dataset. The training objective is cross-entropy loss:

(16) $$L_{cls}=-\sum_{i}y_{i}\log\left({\hat{y}}_{i}\right)$$

where $y_{i}$ is the ground truth label, and ${\hat{y}}_{i}$ is the predicted probability for class *i*.

## 4. Experiments

### 4.1. Datasets and Preprocessing

#### 4.1.1. ABIDE I Dataset

We evaluate our proposed TwoTST framework on the Autism Brain Imaging Data Exchange (ABIDE) I dataset [5], a large-scale multi-site public repository that aggregates resting-state functional magnetic resonance imaging (rs-fMRI) data from 17 international imaging sites. The dataset comprises 1112 subjects (age: 17.0±8.0 years; 948 males and 164 females), including individuals diagnosed with Autism Spectrum Disorder (ASD) and typically developing (TD) controls.

#### 4.1.2. Data Preprocessing

We utilized the preprocessed rs-fMRI data processed via the Configurable Pipeline for the Analysis of Connectomes (CPAC) [28], downloaded from the Preprocessed Connectomes Project. The preprocessing pipeline included slice timing correction, motion correction, nuisance regression (including motion parameters, white matter, and cerebrospinal fluid signals), band-pass filtering (0.01–0.1 Hz), and registration to MNI152 standard space.

The rs-fMRI data were parcellated using the Craddock 200 (CC200) atlas [28], which divides the brain into R=200 spatially coherent and functionally homogeneous regions of interest (ROIs). For each subject, the mean time series within each ROI was extracted, yielding a 2D matrix representation $X\in R^{T\times R}$, where *T* denotes the number of time points (100 in the ABIDE dataset) and R=200 represents the number of ROIs.

#### 4.1.3. Quality Control

After quality control to remove subjects with excessive head motion or incomplete data coverage, we performed additional data cleaning to exclude subjects with any ROI containing all-zero values (indicating missing or corrupted data). This resulted in a final cohort of 963 subjects (493 with ASD, 470 TD controls) for model training and evaluation. The processed data were formatted as a tensor with dimensions 963×100×200 (963 subjects, 100 time points, 200 ROIs).

### 4.2. Experimental Settings

#### 4.2.1. Implementation Details

All experiments were implemented in PyTorch 2.5.1+cu124 and conducted on NVIDIA RTX 4090D GPUs. The dataset was split into training (70%), validation (10%), and test (20%) sets with stratified sampling to maintain class balance.The model and training configurations are shown in Table 2.

#### 4.2.2. Model Evaluation

We evaluated model performance using five metrics: Area Under the ROC Curve (AUC), Accuracy (ACC), Sensitivity (True Positive Rate), Specificity (True Negative Rate), and F1 Score. AUC was used as the primary metric for model selection during training.

### 4.3. Results

#### 4.3.1. Self-Supervised Pre-Training Loss

**Figure 2:**
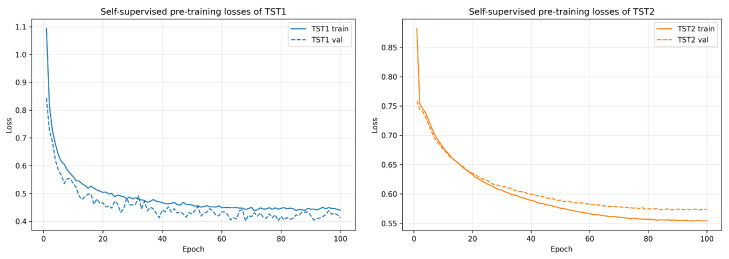
Self-supervised pre-training reconstruction loss.

Figure 2 shows self-supervised pre-training reconstruction loss curves of the two temporal transformers. The left panel shows the training and validation reconstruction losses of TST1 across epochs, and the right panel shows the corresponding losses of TST2. Both models exhibit stable convergence under the self-supervised objective, indicating that the pre-training stage successfully learns meaningful temporal representations.

#### 4.3.2. Effect of Freezing Strategies for Contrastive Learning

**Figure 3:**
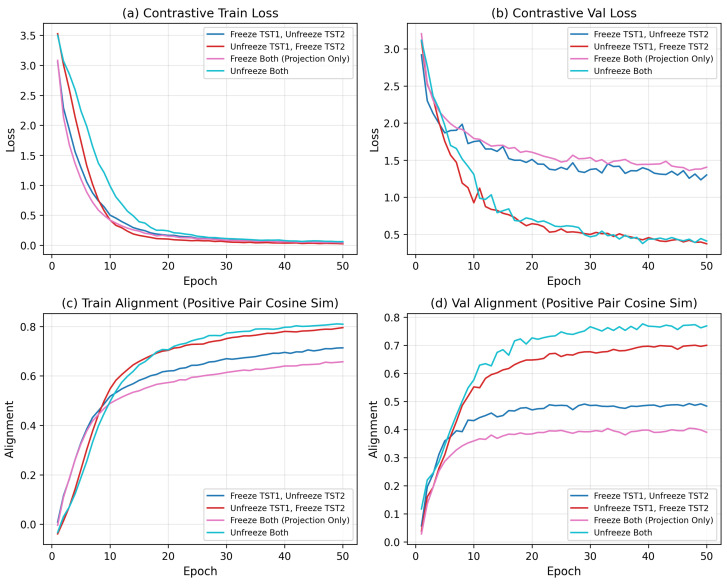
Contrastive pre-training performance under different encoder freezing strategies.

As shown in Figure 3, to quantify how much of the downstream performance comes from updating the backbone encoders versus the projection heads, we compare four freezing strategies during contrastive learning: (i) freeze both TST1 and TST2, (ii) freeze TST1 only, (iii) freeze TST2 only, and (iv)unfreeze both encoders while always training the projection heads. Across all fusion variants, we consistently observe the ordering

unfreezeboth>freezeTST1>freezeTST2>freezeboth,

showing that allowing both temporal and connectivity encoders to adapt to the contrastive objective yields the most discriminative joint representation. However, the performance gap between unfreeze both and freeze TST1 is smaller than the gap to the other two settings, suggesting that the PCC encoder (TST2) is more sensitive to task-specific adaptation.

#### 4.3.3. Comparison of Fusion Strategies

After fixing the best pre-training and contrastive-learning strategy (pre-train TST1/TST2, enable contrastive learning with frozen projection heads at fine-tuning, and unfreeze both encoders), we systematically compare five fusion mechanisms between temporal dynamics (TST1) and functional connectivity (TST2): Concat, Gated fusion, Cross-Attention, Bilinear pooling, and Attention Pooling. For each fusion method, we run five experiments with different random seeds and report the mean and standard deviation of performance on the subject-level test set.

Table 3 summarizes the results (Mean ± Std over 5 runs). Overall, Gated and Concat fusion provide strong baselines, while Attention Pooling achieves competitive performance and is selected as our default fusion in the final configuration due to its simplicity and stability.

To further investigate how much each module adapts during fine-tuning, we measure the mean relative parameter change of TST1, TST2, and the two projection heads across the five seeds. The results are shown in Table 4.

We find that TST1 and TST2 exhibit relatively small changes around 0.32–0.44, while the projection heads show much larger changes around 0.31–1.45, especially under Gated and Attention Pooling fusion. In contrast, under Cross-Attention and Bilinear fusion, the projection heads change less, suggesting that the contrastive representations are already better aligned with the downstream task in these settings. These results support our design choice of using projection-based fusion with a light-weight classifier on top of the pre-trained encoders.

#### 4.3.4. Leave-One-Site-Out (LOSO) Validation

Based on the optimal configuration (Attention Pooling fusion, self-supervised pre-training, contrastive learning, and unfrozen encoders), we conduct **Leave-One-Site-Out (LOSO)** cross-validation across 19 independent scanning sites to verify the cross-site generalization ability of our model. We adopt subject-level evaluation with majority voting to aggregate predictions from different sliding windows, and report AUC, Accuracy, Sensitivity, Specificity, and F1 Score for each site.

Table 5 details the LOSO performance on all 19 sites. The proposed model achieves stable and reliable classification performance across most heterogeneous sites, which demonstrates its strong generalization to multi-center fMRI data and practical clinical value.

Most sites achieve AUC ≥0.70, and UM_2 yields the best performance (AUC = 0.93, Accuracy = 0.88, F1 = 0.91). Sites including CMU, SDSU, and USM also obtain AUC above 0.80. These results confirm that the combination of self-supervised pre-training, contrastive feature alignment, and Attention Pooling fusion effectively alleviates multi-center data distribution shift, enabling robust and accurate ASD identification at a cross-site level.

#### 4.3.5. Explanation Studies

To assess the model interpretability of the proposed TwoTST model, we analyze which functional connections and regions of interest (ROIs) the final classifier actually relies on when predicting ASD. Concretely, we work with the best-performing configuration, load the final checkpoint, and compute gradient-based importance scores with respect to the ASD logit.

Given an input sample *x*, let $f_{\mathrm{ASD}}\left(x\right)$ denote the logit of the ASD class. For the connectivity stream, we consider the vectorized upper triangle of the PCC matrix, i.e., one scalar ${\mathrm{PCC}}_{ij}\left(x\right)$ per connection (i,j). For each sample we compute the gradient

$$\frac{\partial f_{\mathrm{ASD}}\left(x\right)}{\partial {\mathrm{PCC}}_{ij}\left(x\right)},$$

take the absolute value, and then average over all analyzed samples:

$$I_{ij}^{\mathrm{conn}}=E_{x}\left[\left|\frac{\partial f_{\mathrm{ASD}}\left(x\right)}{\partial {\mathrm{PCC}}_{ij}\left(x\right)}\right|\right].$$

The resulting importance matrix $I^{\mathrm{conn}}\in R^{n_{\mathrm{ROI}}\times n_{\mathrm{ROI}}}$ assigns one importance score to each functional connection. A large $I_{ij}^{\mathrm{conn}}$ means that small perturbations of the connection between ROI *i* and ROI *j* cause strong changes in the ASD logit, so the model heavily relies on this edge when distinguishing ASD from typically developing controls (TC).

For the time-series stream, we take the 4D tensor of gradients of $f_{\mathrm{ASD}}\left(x\right)$ w.r.t. the BOLD time series $X\left(x\right)\in R^{T\times n_{\mathrm{ROI}}}$. For each ROI *k*, we first aggregate gradients along the temporal dimension, and then average across samples:

$$I_{k}^{\mathrm{ROI}}=E_{x}\left[\frac{1}{T}\sum_{t=1}^{T}\left|\frac{\partial f_{\mathrm{ASD}}\left(x\right)}{\partial X_{t,k}\left(x\right)}\right|\right].$$

This yields one scalar importance score per ROI. ROIs with larger $I_{k}^{\mathrm{ROI}}$ are those whose temporal dynamics are most influential for the final ASD decision. All indices are defined in the CC200 atlas; during visualization, we map each ROI index to the corresponding anatomical label and lobe/hemisphere information.

**Figure 4:**
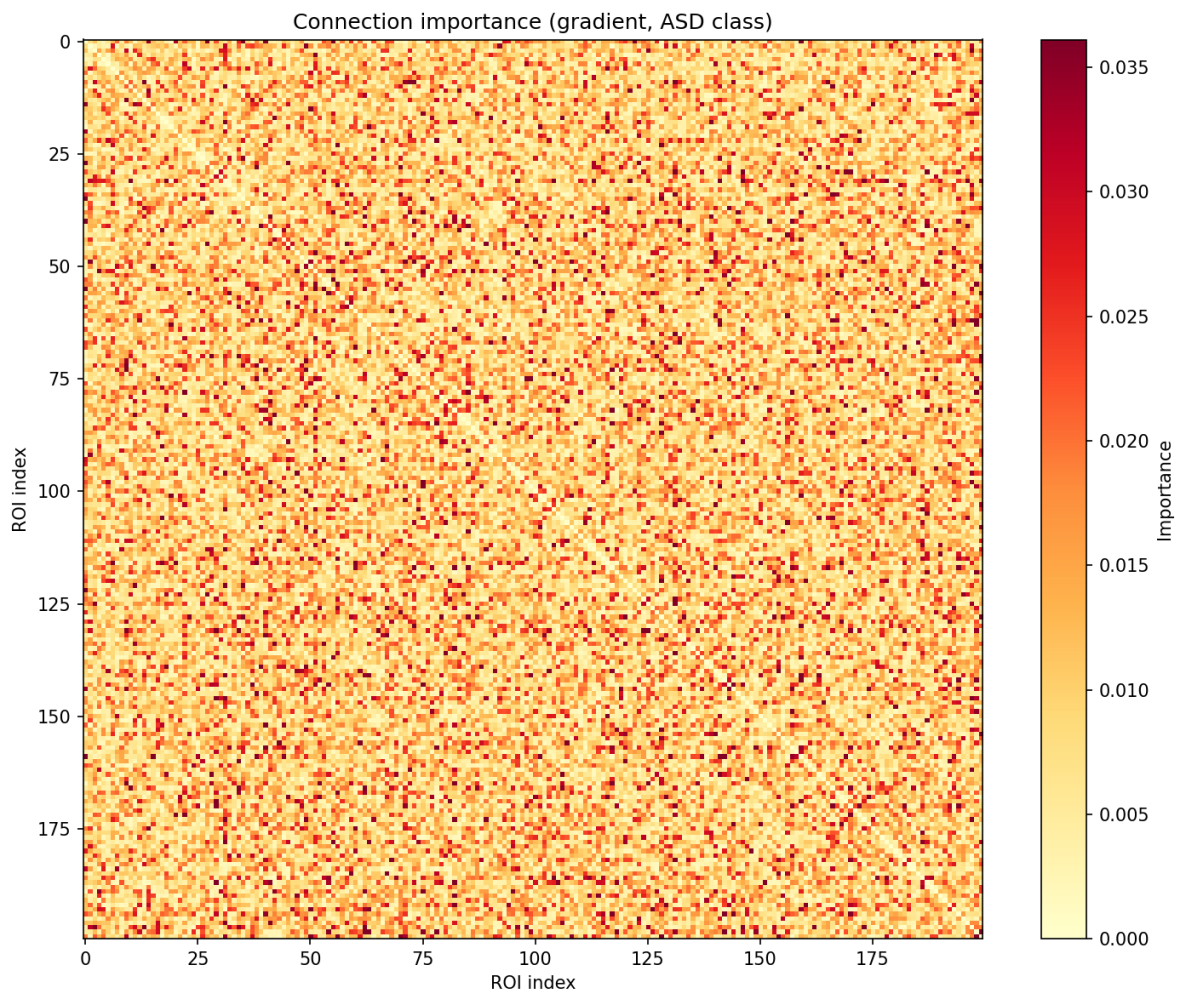
Gradient-based connection importance matrix $I^{\mathrm{conn}}\in R^{200\times 200}$ for the ASD logit.

Figure 4 shows the full 200×200 connection-importance matrix $I^{\mathrm{conn}}$ as a heatmap, revealing a clearly non-uniform pattern: only a relatively sparse subset of connections exhibits high importance scores, while most are close to zero. This indicates that, although the model is trained on all possible pairwise connections, the final decision function effectively concentrates on a small set of discriminative edges, which can be interpreted as candidate abnormal connectionsassociated with ASD.

**Figure 5:**
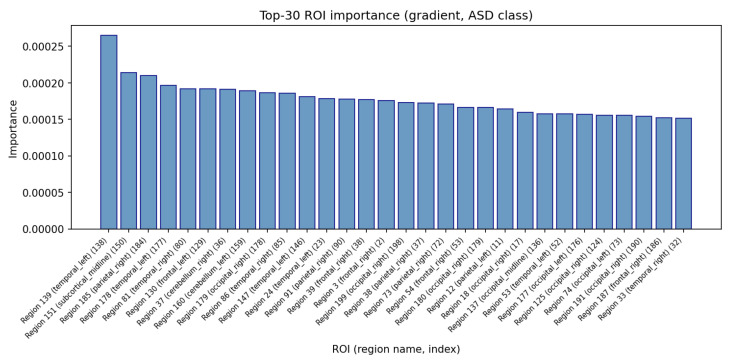
Top ROIs ranked by gradient-based importance $I_{k}^{\mathrm{ROI}}$ for the ASD logit, annotated with CC200 labels.

Figure 5 summarizes the ROI importance vector $I^{\mathrm{ROI}}$ as a bar plot, sorted in descending order and annotated with CC200 region labels. The distribution is again strongly skewed: only a limited number of ROIs contribute substantially to the ASD logit, whereas many regions have negligible influence. From a neuroscientific perspective, these highly weighted ROIs can be viewed as key regions whose BOLD dynamics are most informative for ASD vs. TC discrimination, while the atlas mapping allows us to relate them to known systems in the ABIDE literature.

**Figure 6:**
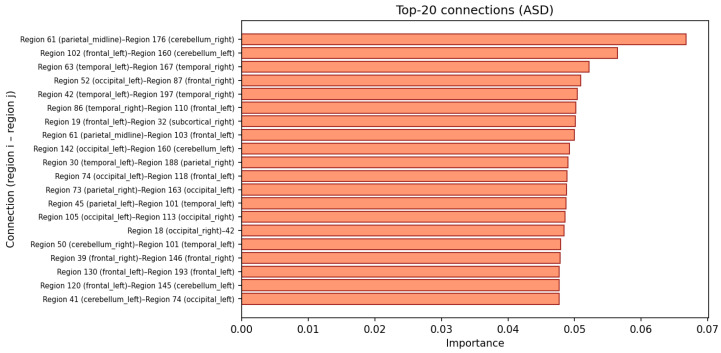
Top functional connections ranked by gradient-based importance $I_{ij}^{\mathrm{conn}}$ for the ASD logit.

Finally, Figure 6 visualizes the top-ranked connections from $I^{\mathrm{conn}}$ as a bar chart of ROI–ROI pairs. Each bar corresponds to a pair (i,j) together with its gradient-based importance score. In combination with group-level PCC differences (ASD vs. TC), these connections can be further characterized as potential under-connected or over-connected edges in ASD. Taken together, the connection-level heatmap, the ROI-level importance distribution, and the top-connection list demonstrate that the model does not behave as an opaque black box: it yields a coherent and spatially structured pattern of important regions and connections, which can be systematically related back to established neuroscientific findings on ASD.

#### 4.3.6. Ablation Studies

We conduct a series of ablation experiments to disentangle the contributions of contrastive learning, pre-training, and fusion choices. All ablation experiments are independently repeated five times with distinct random seeds to ensure reproducibility. For statistical validation, we report the mean AUC (±standard deviation, SD) as the primary metric, and perform paired two-tailed *t*-tests to compare performance between experimental conditions; Bonferroni correction is applied for multiple comparisons to control Type I error. Statistical significance is denoted as: * p<0.05, ** p<0.01, *** p<0.001.

First, we compare configurations with and without contrastive learning under the same fusion and freezing strategy. For the “without” contrastive learning group, we do not utilize the two pre-trained Proj. Head 1 and Proj. Head 2 in the model architecture.

As shown in Table 6, with the exception of Bilinear fusion (non-significant change, p=0.12), all other fusion methods (Concat, Gated, Cross-Attention, and Attention Pooling) achieved a statistically significant AUC improvement when contrastive learning and projection heads were enabled (all p<0.05 after Bonferroni correction). Concat fusion yielded a notably larger and highly significant gain of +0.06 (*** p=0.0003), indicating that contrastive alignment is particularly helpful for more expressive fusion modules.

Second, we perform a “no pre-training” ablation where TST1 and TST2 are randomly initialized and trained end-to-end together with the fusion module. The results are shown in Table 7.

Across multiple fusion types, pre-training consistently improved or preserved performance, with statistically significant gains observed for Gated (+0.02, * p=0.041), Cross-Attention (+0.07, *** p=0.0002), Bilinear (+0.03, ** p=0.008), and Attention Pooling (+0.02, * p=0.039) after Bonferroni correction. Only Concat fusion showed a non-significant small improvement (+0.01, p=0.21). Combined with the parameter-change analysis, these statistically validated results demonstrate that self-supervised pre-training followed by contrastive projection and light-weight fine-tuning is a more effective strategy than purely supervised training from scratch.

Third, we perform a “single-backbone” ablation where we only keep one of the two temporal transformers. Concretely, we consider (i) TST1-only: we pre-train TST1, attach a task-specific prediction head, and fine-tune the whole model end-to-end; and (ii) TST2-only: we pre-train TST2, attach the same prediction head, and fine-tune it in the same way. We then compare these single-backbone models against the full TwoTST model (TST1 + TST2 with Attention Pooling fusion) under identical training and evaluation protocols, using one-way ANOVA with post hoc Bonferroni correction for multiple comparisons. The results are shown in Table 8.

One-way ANOVA reveals a significant main effect of model architecture on AUC (F(2,12)=19.87, p<0.001). Post hoc Bonferroni-corrected comparisons confirmed that the full TwoTST model achieved significantly higher AUC than TST1-only (−0.04, ** p=0.007) and TST2-only (−0.03, * p=0.043). While single pre-trained backbones yielded competitive performance, the statistically validated improvement of TwoTST over the best single-backbone variant (TST2-only) by +0.03 absolute AUC (from 0.71 to 0.74) confirms that jointly leveraging two complementary temporal representations is more effective than relying on a single pre-trained TST. Combined with the pre-training ablations above, this result further supports our design choice of using two pre-trained temporal transformers followed by lightweight fusion and task-specific fine-tuning.

## 5. Discussion

### 5.1. Comparison with Existing Methods

To ensure the rigor and reproducibility of our comparative experiments (addressing generalization and fair comparison concerns), we (1) downloaded the official source code of all baseline methods from their respective paper repositories; (2) re-ran all methods on the **identical ABIDE I (CC200)** sample split (same training/validation/test sets) to eliminate dataset bias; (3) independently repeated each experiment for five distinct random seeds to quantify performance variability; (4) adopted AUC ± standard deviation (SD) as the core evaluation metric (supplemented with ACC, Sensitivity, Specificity ± SD for comprehensive comparison). Table 9 presents a rigorous comparison with traditional machine learning approaches (SVM and Random Forest), deep learning methods (DAE), and graph-based neural networks (MVS-GCN, FBNET, and WL-DEEPGCN), while Table 10 details the consistent implementation settings across all methods.

Our proposed TwoTST framework achieves substantial improvements over all baseline methods. Traditional machine learning methods (SVM and Random Forest) and early deep learning approaches (DAE) achieve relatively modest performance (accuracy around 67–68%), primarily because they rely on handcrafted features or shallow representations that fail to capture the complex temporal dynamics and connectivity patterns in fMRI data. Graph-based methods (MVS-GCN and FBNET) show improved performance by explicitly modeling brain network topology, but they typically focus on static connectivity matrices and do not fully exploit the temporal information inherent in fMRI time series.

**Computational Complexity, Training Time, and Scalability.** To provide a complete comparison, we report model size, approximate training time, and scalability characteristics in Table 11. TwoTST consists of two Transformer encoders (TST1 for time series, TST2 for connectivity), projection heads for contrastive learning, and an Attention Pooling fusion module, yielding approximately 36.5 M parameters. Traditional methods (SVM and Random Forest) have negligible parameters and train in minutes but rely on handcrafted features. DAE and graph-based methods (MVS-GCN and FBNET) use 1–3 M parameters and typically require 5–10 min of GPU training on ABIDE. TwoTST involves a multi-stage pipeline: pre-training TST1 and TST2, contrastive learning, and fine-tuning, totaling approximately 20–30 min on a single GPU (e.g., NVIDIA RTX 4090D) for the full workflow on ABIDE I. Despite a higher parameter count and longer training time, TwoTST achieves the best performance and offers favorable scalability: (i) inference is linear in the number of samples and sequence length; (ii) the dual-stream design allows independent scaling of temporal (TST1) and connectivity (TST2) capacities; (iii) the architecture generalizes to other atlases by adjusting n_rois and pcc_dim, with complexity scaling as $O(n_{\mathrm{ROI}}^{2})$ for the connectivity stream. For deployment, only the fine-tuned model is required; pre-training needs to be performed once per dataset.

### 5.2. Limitations and Future Directions

Despite the promising results, our study has several limitations that warrant further investigation:

**Single Dataset Evaluation:** Our experiments were conducted exclusively on the ABIDE I dataset. While this enables direct comparison with existing methods, the generalizability of our approach to other ASD datasets or different neurological conditions remains to be validated. Future work should evaluate TwoTST on additional datasets such as ABIDE II and other multi-site repositories.

**Fixed Parcellation:** We used the CC200 atlas with 200 ROIs throughout our experiments. Different parcellation schemes (e.g., AAL and Schaefer) or finer-grained parcellations may yield different results. Investigating the impact of parcellation choice on model performance is an important direction for future research.

**Multi-modal Integration:** Our current framework focuses solely on rs-fMRI data. Integrating structural MRI (sMRI), diffusion tensor imaging (DTI), or clinical phenotypic information could potentially improve diagnostic accuracy and provide a more comprehensive characterization of ASD.

In summary, TwoTST represents a significant advancement in fMRI-based ASD classification by effectively combining temporal dynamics and functional connectivity through a dual-stream Transformer architecture. The substantial performance improvements over existing methods demonstrate the potential of self-supervised contrastive learning and Attention Pooling fusion for neuroimaging analysis.

## 6. Conclusions

This paper presents TwoTST, a dual-stream Transformer framework for fMRI-based ASD classification that simultaneously captures temporal dynamics (via TST1 processing raw ROI time series) and global connectivity patterns (via TST2 processing PCC matrices). We perform masked reconstruction pre-training and contrastive learning to align dual-branch features. Fine-tuning experiments show that unfreezing both encoders with contrastive projection heads achieves optimal performance. Among five fusion methods over five random seeds, Attention Pooling performs best with an AUC of 0.74±0.04. LOSO cross-validation proves its strong cross-site generalization. The pre-trained encoders are updated moderately (0.32–0.44) in fine-tuning, supporting the effectiveness of pre-training. Gradient-based importance scores and connection-importance matrix offer interpretable ASD biomarkers. Comprehensive ablation experiments validate each key component and the whole TwoTST framework.

## 7. Patents

The authors declare that no patents resulted from the work reported in this manuscript.

## Figures and Tables

**Table 1:** Five residual connect fusion methods. Bold indicates the optimal method.

Method	Key Components
Concat	Concat, MLP
Gated	Concat, Gate Network, Weighted fusion, MLP
Cross-Attention	Cross-attention Concat, MLP
Bilinear	Bilinear layer, Concat, MLP
**Attention Pooling**	**Stack**, **Attention network**, **Weighted fusion**, **MLP**

**Table 2:** Model and training configurations.

Configuration	Value
TST1 (ROI Transformer)	
Embedding dimension ($d_{1}$)	512
Number of layers ($L_{1}$)	6
Attention heads	8
Feed-forward dimension	2048
Dropout rate	0.1
TST2 (PCC Transformer)	
Model dimension ($d_{2}$)	256
Number of layers ($L_{2}$)	2
Attention heads	8
Feed-forward dimension	512
Dropout rate	0.1
PCC vector dimension	19,900
Pre-training	
Epochs	100
Loss function	MSE
TST1 mask ratio	0.25–0.5 (random)
TST2 mask ratio	0.15
Optimizer	Adam
Learning rate	$1\times 10^{-4}$
Weight decay	$1\times 10^{-4}$
Batch size	32
Contrastive epochs	50
Contrastive loss	InfoNCE
Contrastive batch size	32
Contrastive learning rate	$1\times 10^{-4}$
Contrastive weight decay	$1\times 10^{-4}$
Temperature (τ)	0.07
Projection hidden dimension	256
Projection output dimension	128
Fine-tuning	
Epochs	100
Early stopping patience	20
Optimizer	Adam
Learning rate	$5\times 10^{-5}$
Weight decay	$1\times 10^{-4}$
MLP hidden dimensions	[256, 64, 2]
Dropout rate	0.3

**Table 3:** Subject-level classification performance of different fusion strategies (5 seeds, Mean ± Std).

Fusion Method	AUC	ACC	Sensitivity	Specificity	F1
Concat	0.71±0.06	0.65±0.04	0.67±0.07	0.63±0.06	0.66±0.05
Gated	0.72±0.05	0.66±0.03	0.68±0.12	0.63±0.18	0.67±0.03
Cross-Attention	0.71±0.04	0.66±0.04	0.63±0.08	0.69±0.05	0.66±0.05
Bilinear	0.60±0.03	0.58±0.03	0.65±0.06	0.50±0.06	0.62±0.03
**Attention Pooling**	0.74±0.04	0.73±0.03	0.70±0.08	0.71±0.10	0.73±0.03

Bold text indicates the optimal fusion method with the best classification performance.

**Table 4:** Mean relative parameter change (Mean ± Std over 5 seeds) for each module under different fusion strategies.

Fusion	TST1	TST2	Proj. Head 1	Proj. Head 2
Concat	0.40±0.04	0.44±0.02	0.96±0.23	0.59±0.03
Gated	0.41±0.04	0.44±0.0074	1.5±0.33	0.66±0.03
Cross-Attention	0.34±0.016	0.39±0.021	0.58±0.17	0.33±0.022
Bilinear	0.33±0.022	0.38±0.031	0.74±0.11	0.31±0.010
Attention Pooling	0.38±0.047	0.41±0.018	1.3±0.66	0.58±0.044

**Table 5:** The performance metrics of each site under LOSO cross-site evaluation for the Attention Pooling fusion approach.

Site	AUC	Accuracy	Sensitivity	Specificity	F1
CALTECH	0.61	0.62	0.63	0.72	0.53
CMU	0.83	0.71	0.75	0.67	0.75
KKI	0.75	0.66	0.4	0.85	0.78
LEUVEN_1	0.76	0.66	0.93	0.36	0.74
LEUVEN_2	0.62	0.53	0.77	0.27	0.63
MAX_MUN	0.61	0.63	0.79	0.25	0.65
NYU	0.73	0.68	0.76	0.59	0.73
OLIN	0.74	0.72	0.69	0.71	0.73
PITT	0.68	0.64	0.62	0.66	0.62
SBL	0.63	0.60	0.58	0.57	0.61
SDSU	0.84	0.59	0.33	1.00	0.50
STANFORD	0.60	0.56	0.55	0.58	0.56
TRINITY	0.73	0.71	0.70	0.73	0.71
UCLA_1	0.70	0.63	0.52	0.71	0.54
UCLA_2	0.76	0.77	0.77	0.77	0.77
UM_1	0.72	0.64	0.62	0.66	0.64
UM_2	0.93	0.88	0.91	0.85	0.91
USM	0.81	0.76	0.68	0.80	0.67
YALE	0.76	0.67	0.68	0.67	0.68

**Table 6:** Performance comparison of 5 fusion methods (with/without contrastive learning, unfrozen strategy).

Fusion Method	Contrastive Learning (Mean AUC ± SD)		ΔMean AUC	Stat. Signif.
	With	Without	(With–Without)	
Concat	0.73±0.02	0.67±0.02	**+0.06**	*** p=0.0003
Gated	0.72±0.01	0.69±0.02	**+0.03**	* p=0.038
Cross-Attention	0.72±0.02	0.69±0.01	**+0.03**	* p=0.042
Bilinear	0.70±0.02	0.72±0.01	**−0.02**	p=0.12 (ns)
Attention Pooling	0.74±0.04	0.71±0.02	**+0.03**	* p=0.045

ns: not significant; * p<0.05, *** p<0.001 (Bonferroni-corrected paired *t*-test). Bold text indicates key experimental data reflecting performance improvements in the contrastive learning comparison.

**Table 7:** Effect of pre-training for TST1 and TST2 (with/without pre-training).

Fusion Method	Pre-Training (Mean AUC ± SD)		ΔMean AUC	Stat. Signif.
	With	Without	(With–Without)	
Concat	0.73±0.01	0.72±0.01	**+0.01**	p=0.21 (ns)
Gated	0.71±0.02	0.69±0.02	**+0.02**	* p=0.041
Cross-Attention	0.73±0.02	0.66±0.03	**+0.07**	*** p=0.0002
Bilinear	0.72±0.01	0.69±0.02	**+0.03**	** p=0.008
Attention Pooling	0.74±0.04	0.72±0.02	**+0.02**	* p=0.039

ns: not significant; * p<0.05, ** p<0.01, *** p<0.001 (Bonferroni-corrected paired *t*-test). Bold text indicates data on the magnitude of performance improvement in the comparison of TST initialization with/without the pre-training stage.

**Table 8:** Effect of using a single pre-trained TST backbone vs. the full TwoTST model.

Model	Test Mean AUC ± SD	ΔMean AUC	Stat. Signif.
TST1-only (pre-train + fine-tune)	0.70±0.02	−0.04	** p=0.007
TST2-only (pre-train + fine-tune)	0.71±0.01	−0.03	* p=0.043
TwoTST (TST1 + TST2, attention pooling)	0.74±0.04	0	–

* p<0.05, ** p<0.01 (ANOVA + post hoc Bonferroni correction). Bold text indicates the maximum value of Test Mean AUC ± SD among the compared models.

**Table 9:** Performance comparison with existing methods on ABIDE I (CC200) (all results are mean ± SD over 5 random seeds).

Method	ACC	Sensitivity	Specificity	AUC
SVM [17]	67.5±2.1	68.7±3.2	57.5±2.8	67.4±2.5
Random Forest [29]	68.0±1.8	69.1±2.9	58.5±3.1	60.8±2.3
DAE [9]	67.6±2.4	69.8±3.5	63.0±2.7	64.5±2.6
MVS-GCN [30]	69.4±2.2	70.5±3.0	64.1±2.9	66.5±2.4
FBNET [31]	70.9±2.5	74.3±3.3	70.8±3.2	72.5±2.8
**TwoTST (Ours)**	73.0±3.0	70.0±8.0	71.0±10.0	74.0±4.0

Bold text indicates the performance data of the TwoTST model proposed in this paper.

**Table 10:** Implementation details comparison of different methods on ABIDE I (consistent settings for fair comparison).

Method	Atlas	FC Generation	Network Architecture
SVM [17]	CC200	PCC	Linear/RBF kernel SVM
Random Forest [29]	CC200	PCC	Random Forest ensemble
DAE [9]	CC200	PCC	Stacked DAE + MLP/CNN
MVS-GCN [30]	CC200	PCC	GCN with multi-view learning
FBNET [31]	CC200	ROIs	Graph generator + GNN
**TwoTST (Ours)**	**CC200**	**ROIs + PCC**	**Dual TST + Attention pooling fusion**

Bold text indicates the implementation components of the TwoTST model proposed in this paper.

**Table 11:** Computational complexity, training time, and scalability comparison on ABIDE I (CC200). Training time is approximate on a single NVIDIA RTX 4090D GPU.

Method	Parameters	Training Time	Scalability
SVM	∼0	∼5 min	$O(n^{2})$ kernel; poor for large *n*
Random Forest	∼0.5 M	∼3 min	O(nlogn) per tree; parallelizable
DAE	∼1–2 M	∼8 min	Linear in *n* per epoch
MVS-GCN	∼1–2 M	∼10 min	Linear in |V|+|E|; graph-dependent
FBNET	∼1–3 M	∼10 min	Linear in graph size
**TwoTST (Ours)**	∼**36.5 M**	∼**20–30 min**	Linear inference; $O(n_{\mathrm{ROI}}^{2})$ connectivity stream

Bold text indicates the parameter scale and training time of the TwoTST.

## Data Availability

The data presented in this study are openly available in https://fcon_1000.projects.nitrc.org/indi/abide/abide_I.html, (accessed on 10 March 2025).
